# Proteomics and phosphoproteomics of freshwater mollusk carcasses reveal novel insights as potential food source

**DOI:** 10.3389/fcimb.2025.1568349

**Published:** 2025-08-14

**Authors:** Gongzhen Liu, Shengkang Wang, Tielin Wang, Changan Zhu, Changlong Li, Xinyi Zhang

**Affiliations:** College of Agriculture and Forestry, Linyi University, Linyi, Shandong, China

**Keywords:** freshwater, mollusks carcasses, proteomics, phosphoproteomics, cellular components, molecular functions, biological processes, differentially expressed proteins (DEPs)

## Abstract

*Cipangopaludina chinensis*, *Radix auricularia*, and *Nodularia douglasiae* are common freshwater mollusks widely distributed in lakes and rivers across China. In this study, (DIA) Data - independent acquisition method was used to analyze the proteomics and phosphoproteomics among three species. The results showed that a total of 1,382 proteins were identified from mollusk carcasses, with 492 proteins were quantitively analyzed. These proteins are mainly involved in amino acid nutrition and metabolism, including glutamate dehydrogenase, glyceraldehyde-3-phosphate dehydrogenase, and tyrosinase copper-binding domain-containing protein. Bioinformatics analysis revealed that the identified proteins are primarily involved in carbon metabolism, microbial metabolism, ubiquitin-mediated proteolysis, and RAS signaling pathways. Notably, this study offers valuable insights into the differential protein expression of proteins across species. Furthermore, proteomics analysis revealed several novel proteins, which helps to improve our understanding of amino acid metabolism, protein diversity, and signaling pathways in these freshwater mollusks.

## 1 Introduction

*Cipangopaludina chinensis* (*C. chinensis*), *Radix auricularia* (*R.auricularia*), and *Nodularia douglasiae* (*N.douglasiae*) are three freshwater mollusk species widely distributed across Asian countries, including China, Japan, and Korea, as well as other parts of the world (Zhou et al., 2022a, Zhou et al., 2022a; Zhao et al, 2022; Choi et al., 2020; Schell et al., 2017). Mollusks inhabit a variety of freshwater environments such as rivers, lakes, rice fields, swamps, and ditches (Marrone et al., 2019). Taxonomically, both *C. chinensis* and *R.auricularia* belong to the phylum Mollusca and class Gastropoda; however, *C. chinensis* is classified under the family Viviparidae, while *R.auricularia* belongs to the family Lymnaeidae (Juhász and Lawton, 2022). In contrast, *N.douglasiae* belongs to the phylum Mollusca, class Bivalvia and family Unionidae, which is very similar to another relative species, *Nodularia breviconcha* (*N.breviconcha*) (Kim et al., 2021; Choi et al., 2020).

Aquatic mollusks play a vital role in ecosystems, significantly influencing food webs and nutrient cycling (Darrigran et al., 2025; Vasta et al., 2015). Economically, they contribute positively to aquaculture by serving as a food source and providing protein-rich nutrition (such as Viviparidae) (Liu et al., 2024), but they can also cause substantial economic losses through macrofouling and pose health risks, particularly in cases involving non-native species (Li et al., 2013; Darrigran et al., 2025). Freshwater mollusks are further recognized as key water quality indicators, natural biofilters, and agents of reproduction and dispersal. First, mollusks are an important part of the food chain in aquatic ecosystems by feeding on aquatic plants, plankton, organic detritus, and dead organisms (Deidda et al., 2021). Second, their sensitivity to environmental changes makes them effective bioindicators for monitoring water quality, including nutrient levels, pollution, and oxygen content (Elder and Collins, 1991; Mienis and Ashkenazi, 2011; Fernandes et al., 2011). Third, mollusks have a strong filtration capacity, enabling them to remove plankton and organic debris from water while metabolizing harmful substances and improving water quality (Giansante and Pelini, 2007). Additionally, they can form intricate symbiotic relationships with other aquatic organisms, enhancing the ecological stability of aquatic ecosystems (Chakraborty and Joy, 2020; de la Ballina et al., 2022). Finally, mollusks contribute to biodiversity by acting as dispersers and reproductive agents, spreading genetic material across diverse habitats and promoting species diversity (Wayne, 2001; Beirão et al., 2019; Gaudin-Zatylny et al., 2022).

*C. Chinensis* carcasses are an important food source for both humans and animals, and several recent studies have explored this species in greater depth. Single-molecule real-time (SMRT) sequencing technology has been used to analyze its full-length transcriptome, revealing that metabolic molecules, signal transduction proteins, and immune-related proteins are involved in drug metabolism and immune response (Zhou et al., 2022a). Meanwhile, high levels of aryl sulfatase and β-glucuronidase were identified in *C. chinensis*, both of which play a key role in the cleavage process of bound natural estrogens (C-NEs) (Zhao et al., 2022). Notably, a comparative analysis of shell proteins between *Pomacea canaliculata* (*P. canaliculata*) and *C. chinensis* showed that carbonic anhydrase was absent in both species, suggesting that freshwater snails may have a unique mechanism for regulating shell calcification and mineralization (Liu et al., 2023).

*R. auricularia* poses potential health risks, as it serves as an intermediate host for zoonotic trematodes. However, *Radix auricularia* also plays an important ecological role in aquatic sphere. *Radix auricularia* is an integral part of freshwater ecosystems, contributing to the balance of the food web, water quality maintenance, and the creation and modification of habitats. Its ecological role highlights the importance of conserving these organisms and their habitats for the overall health and functioning of freshwater environments. These include *Clinostomum complanatum* (Chung et al., 1998), *Trichobilharzia franki* (Juhász et al., 2022), and *Petasiger exaeretus* (Bashe and Ali, 2019). The draft genome of the pulmonate freshwater snail *R.auricularia* has been sequenced, providing a valuable foundation for genomic and population genetics research (Schell et al., 2017). Furthermore, phylogenetic analyses exploring the evolution of Toll-like receptors (TLRs) in this species revealed that, unlike many other mollusks, R. auricularia also possesses class I TLRs (Juhász et al., 2022).

*N*.*douglasiae* is a freshwater bivalve mollusk with distinct morphological characteristics that differs significantly from *C. chinensis* and *R.auricularia.* Investigating the genetic structure and diversity of N. douglasiae from the Yangtze River basin in China has provided important insights for the conservation of genetic diversity and effective management of riverine ecosystems (Liu et al., 2017). Additionally, the complete mitochondrial genomes of *N.douglasiae* and *N.nipponensis* have been sequenced from the Lake Biwa system in Japan (Mabuchi et al., 2021). Mitochondrial *CO1 and 16S rRNA* gene sequences from *N. douglasiae* have also been used to analyze phylogenetic relationships and population genetic structure, revealing close genetic similarity to *N.breviconcha* from the Korean Peninsula (Choi et al., 2020; Kim et al., 2021). Recent studies have further demonstrated that the kidneys of *N.douglasiae* improve water excretion efficiency in freshwater mussels through counter-current systems (Nikishchenko et al., 2022).

Although several studies have examined these three freshwater species, little research has been conducted at the protein level. The carcasses of all three species contain abundant proteins and proteases with potential for future applications. In this study, we aimed to analyze proteomic profile differences in mollusk carcasses among three species. Our results reveal novel proteins and differentially expressed proteins (DEPs) across the three species, offering valuable insights into protein composition, nutritional potential, and involvement in key signaling pathways.

## 2 Materials and methods

### 2.1 Samples

The three species (*Cipangopaludina chinensis*, *Radix auricularia*, and *Nodularia douglasiae*) were initially collected from the Yi River Basin in Shandong Province, China (latitude 34°22′–36°13′ N; longitude 117°24′–119°11′ E), a region characterized by a temperate monsoon climate.). We choose the same season in clean waters areas for three species. We used *C.chinensis* (2.8cm×1.5cm)*, R.auricularia* (1.5cm×0.5cm) and *N.douglasia* (6.0cm×1.5cm) carcasses (muscle) respectively as protein samples for proteomic and phosphoproteomics analysis. All protein samples were taken from the same part of the carcass, after postmortem examination immediately transported on dry ice. The experimental groups were designated as Group A (*C. chinensis*), Group B (*R. auricularia*), and Group C (*N. douglasiae*), with each individual treated as a separate biological replicate.

### 2.2 High -performance liquid chromatography fractionation

Samples were concentrated, digested, and extracted, and the protein concentrations were determined using the Bradford assay. Protein sample preparation followed methods described previously (Liu et al., 2022).

### 2.3 LC−MS/MS analysis

For construction of the transition library, shotgun proteomics was performed using an Ultimate 3000 Ultra High-Performance Liquid Chromatography (UHPLC) system (Dionex) coupled with a Q Exactive HF mass spectrometer (Thermo Fisher Scientific). The detailed procedures were based on previously established protocols (Liu et al., 2022).

### 2.4 Phosphorylation analysis

For phosphopeptide enrichment, an appropriate volume of PuriMag Si-TiO_2_ magnetic beads was added to a 1.5 mL tube and washed three times with sample buffer (1 M glycolic acid in 80% acetonitrile [ACN] and 5% trifluoroacetic acid [TFA]). The sample buffer was then added and thoroughly mixed, followed by incubation at room temperature for 20 minutes. After incubation, the supernatant was transferred to a new tube. The beads were washed once with a sample buffer, twice with washing buffer (80% ACN, 1% TFA), and twice with an additional buffer (10% ACN, 0.2% TFA). Phosphopeptides were eluted with 5% ammonium hydroxide (NH_4_OH) and acidified with formic acid.

A second enrichment was performed using immobilized metal affinity chromatography (IMAC). IMAC beads (200 uL) were transferred to a 1.5 mL tube, centrifuged at 2,100 ×g for 30 seconds, and washed with 6% acetic acid. The beads were incubated in 0.1 M FeCl_3_ in 6% acetic acid for 2 hours, then washed three times with 6% acetic acid. A mixed buffer (250 mM acetic acid, 30% ACN) was added to a 50% slurry. The dried peptide sample was resuspended in this slurry at a concentration of 1 μg/μL and incubated at room temperature with vigorous shaking for 1 hour. After incubation, the supernatant was transferred to a new tube. The beads were washed twice with buffer and once with water. Phosphopeptides were then eluted with 5% NH_4_OH and acidified with formic acid. All enriched samples were desalted and vacuum freeze-dried. Finally, phosphopeptides enriched using PuriMag Si-TiO_2_ and IMAC were pooled and desalted.

### 2.5 Bioinformatic analysis

The identified proteins were classified using Gene Ontology (GO) annotation. The GO annotation proteome was derived from the UniProt-GOA database (http://www.ebi.ac.uk/GOA/). The Kyoto Encyclopedia of Genes and Genomes (KEGG) database was used to annotate protein pathways. KEGG pathway mapping was carried out using the KEGG Automatic Annotation Server (KAAS).

## 3 Results

### 3.1 Identified proteins

Each individual carcass of *C. chinensis*, *R. auricularia*, and *N. douglasiae* was analyzed using proteomic and phosphoproteomic methods. phosphoproteomics. Cellular component (CC), molecular function (MF), and biological process (BP)—as well as Kyoto Encyclopedia of Genes and Genomes (KEGG) pathway analysis, were used to examine protein expression differences among the three pairwise comparisons: Avs-B, Avs-C and Bvs-C.

**Figure 1:**
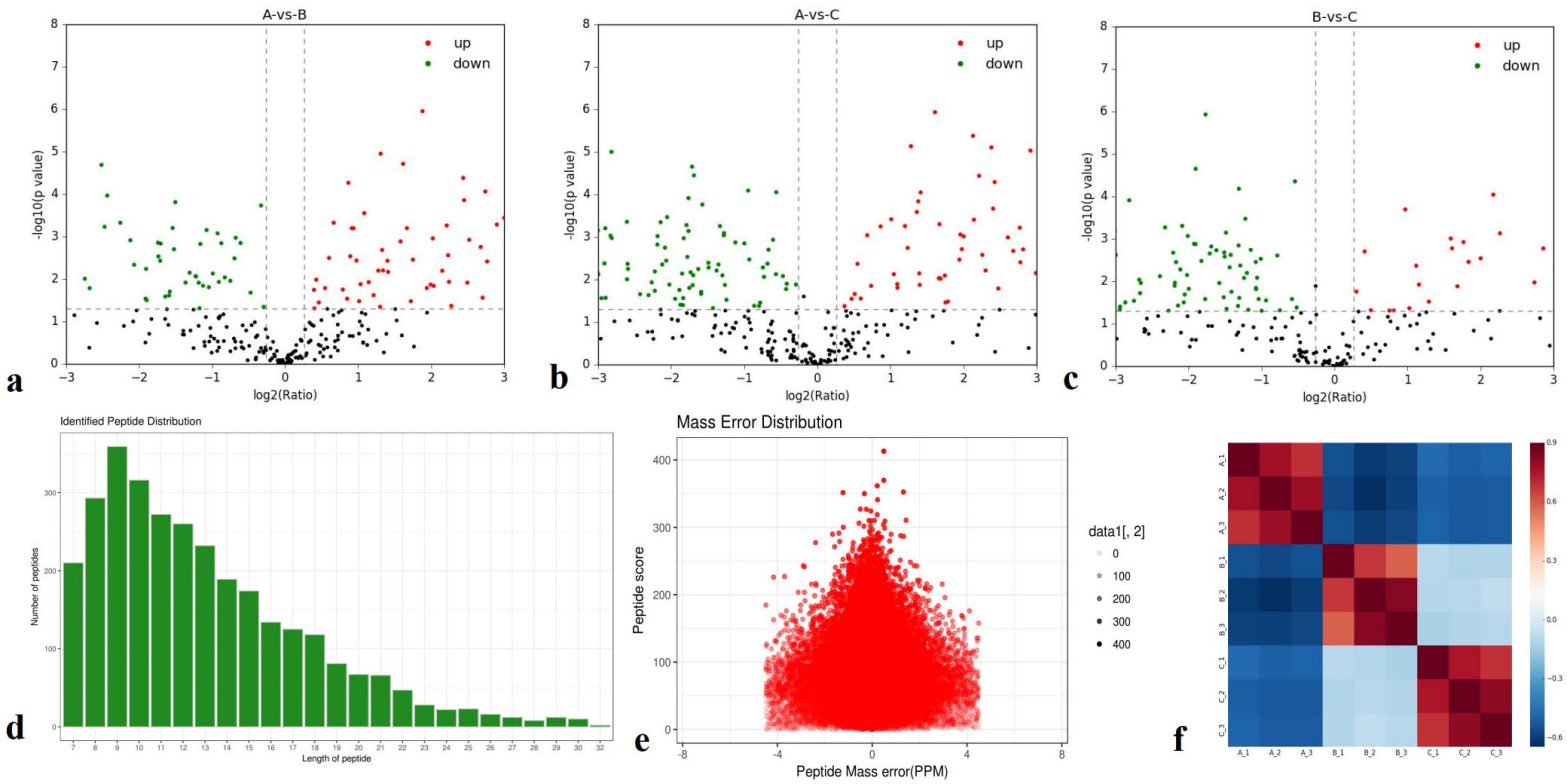
Total identified proteins. **(a-c)** Protein expression ratios of A-vs-B, A-vs-C, and B-vs-C; **(d)** Distribution of identified peptides; **(e)** Mass error distribution; **(f)** Repeatability analysis across groups A-vs-B, A-vs-C, and B-vs-C.

A total of 1,382 proteins were identified, and 492 were quantitatively analyzed. Among these, 100 (62), 66 (101), and 38 (98) significantly upregulated (downregulated) differentially expressed proteins (DEPs) were detected in pairwise comparisons (A-vs-B, A-vs-C, and B-vs-C) (**Figures 1a–f**).

### 3.2 Functional classification of DEPs

**Figure 2:**
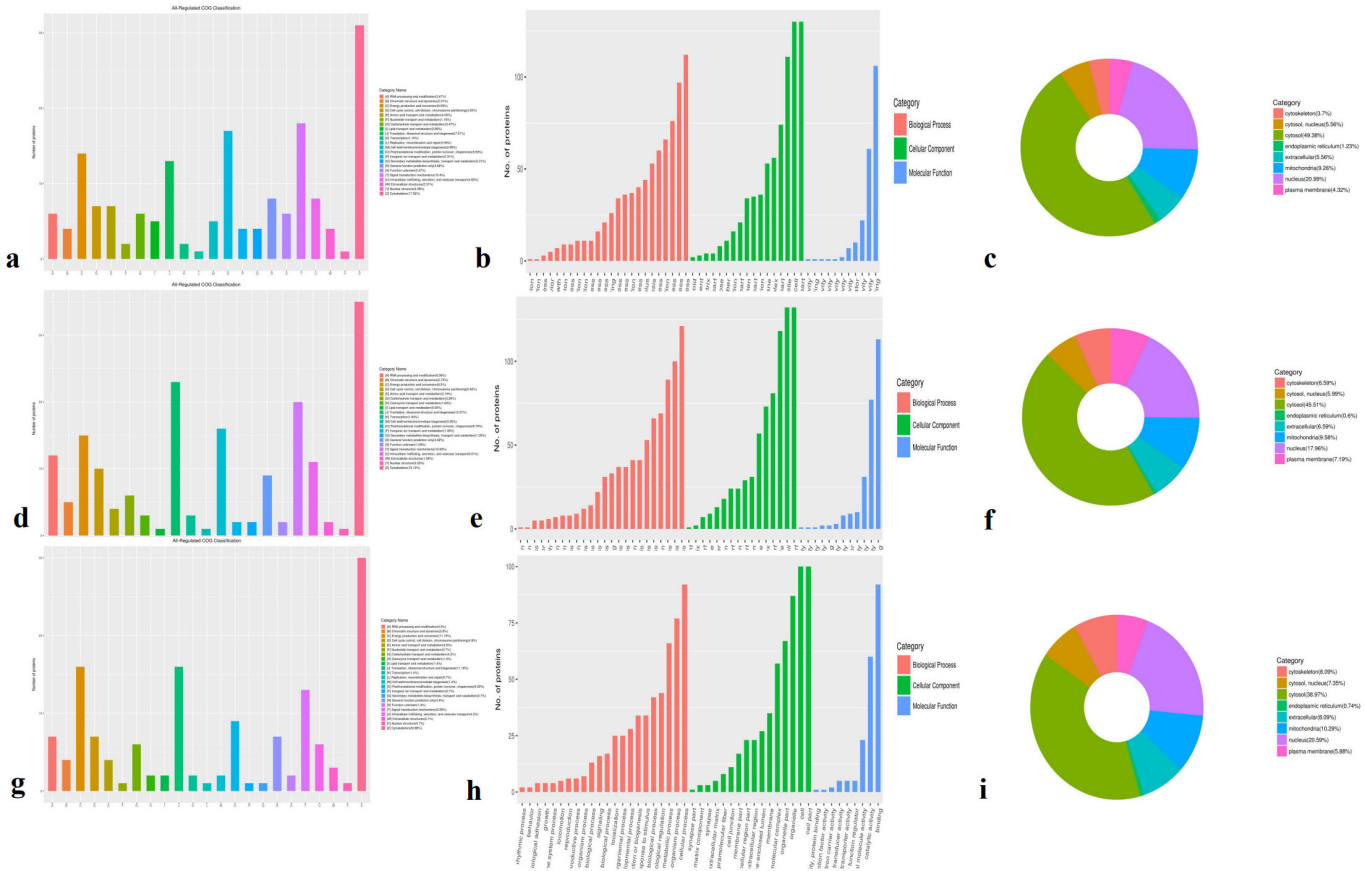
GO classification involved in cellular components, molecular functions, biological processes and subcellular location in three species. **(a, d, g)** All regulated classification in A -vs- B, A -vs- C, B -vs- C, respectively; **(b, e, h)** The category DEPs in cellular components, molecular function, biological processes in A -vs- B, A -vs- C, B -vs- C, respectively; **(c, f, i)** The DEPs subcellular location in A -vs- B, A -vs- C, B -vs- C, respectively. GO Annotation: Gene Ontology (GO) is a major bioinformatics initiative to unify the representation of gene and gene product attributes across all species.

DEPs were defined based on a protein quantification threshold of fold change ≥ 1.2 and p-value ≤ 0.05. Functional classification was conducted for all regulated proteins, including assessments of cellular components, molecular functions, biological processes, and subcellular localization. Across all three comparisons (A-vs-B, A-vs-C, and B-vs-C), the DEPs were primarily classified into the following functional categories: cytoskeleton (17.92%–20.98%), ribosomal structure and biogenesis (11.19%–12.57%), signal transduction mechanisms (9.09%–10.93%), energy production and conversion (8.09%–11.19%), and osttranslational modification, protein turnover, and chaperones (6.29%–9.83%) (**Figures 2a, d, g**). Functional classification revealed clear differences among the three comparison groups. DEPs were predominantly involved in cellular components such as cells, cell parts, organelles, membranes, macromolecular complexes, and cell junctions (shown in green in **Figures 2b, e, h**). In terms of molecular function, these proteins were associated with binding, catalytic activity, and structural molecular activity (blue columns in **Figures 2c, f, i**). Biological process analysis showed that DEPs were involved in cellular processes, single-organism processes, metabolic processes, localization, regulation of biological processes (both positive and negative), response to stimuli, and overall biological regulation (orange column in **Figure 2**). Subcellular localization results indicated that most DEPs were concentrated in the cytosol (38.97%–49.38%), nucleus (17.96%–20.99%), and mitochondria (9.26%–10.29%). Notably, cytosolic proteins exhibited a nearly 10% difference in abundance across the three comparisons.

### 3.3 Functional enrichment of DEPs

In contrast to the functional classification, a functional enrichment analysis of all 1,260 proteins highlighted distinct differences in protein domains, biological processes, and KEGG pathways across the three comparison groups.

**Figure 3:**
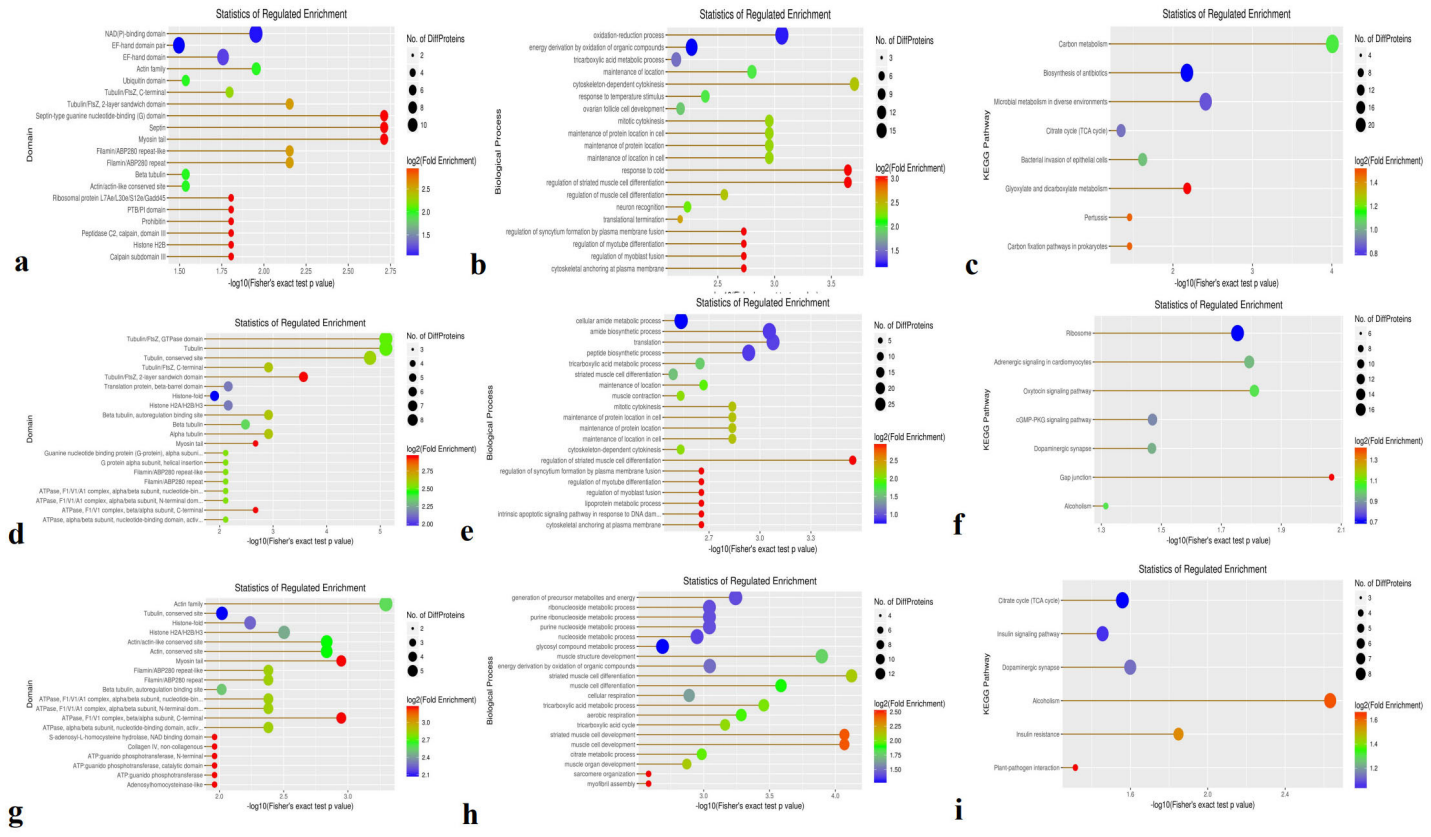
The functional enrichment of DEPs is involved in domains, biological processes, and KEGG in three species. **(a, d, g)** Enrichment domains in A -vs- B, A -vs- C, and B -vs- C, respectively; **(b, e, h)** Enrichment of biological processes in A -vs- B, A -vs- C, and B -vs- C, respectively; **(c, f, i)** Enrichment of KEGG in A -vs- B, A -vs- C, B -vs- C, respectively.

Domain enrichment analysis revealed 158 DEPs in the Avs. B group, including 97 upregulated and 61 downregulated proteins. The majority of enriched domains were NAD(P)-binding domains and EF-hand domain EF-hand domains (**Figure 3a**). In the Avs. C group, 163 DEPs were identified—64 upregulated and 99 downregulated (**Figure 3d**) Tubulin—with enrichment in domains related to tubulin, including the Tubulin (FtsZ, GTPase) domain Tubulin (FtsZ, GTPase domain) and Tubulin (conserved site)conserved tubulin sites. In the Bvs. C group, 132 DEPs were detected (35 upregulated and 97 downregulated), with actin family domains being the most enriched (**Figure 3g**). actin family. Notably, the upregulated and downregulated DEPs exhibited distinct domain patterns across the three comparisons.

Biological process enrichment analysis showed that oxidation-reduction processes and energy derivation from the oxidation of organic compounds in the A-vs.-B group (**Figure 3b**). Compared with the A-vs-B group, the majority of DEPs in the A-vs-C group were involved in the cellular amide metabolic process, translation, amide biosynthetic process, and peptide biosynthetic process (**Figure 3e**). In the B-vs-C group, DEPs were primarily associated with the generation of precursor metabolites and energy, including the metabolism of glycosyl compounds, nucleosides, purines, ribonucleosides, and purine ribonucleosides, as well as energy derivation through the oxidation of organic compounds and muscle structure development (**Figure 3h**).

KEGG pathway enrichment analysis identified 414 proteins, with 85 DEPs in the A-vs,-B group—52 upregulated and 33 downregulated (**Figure 3c**). These DEPs were mainly associated with carbon metabolism, microbial metabolism in diverse environments, and the biosynthesis of antibiotics. In the A-vs-C group, 102 DEPs were found (36 upregulated and 66 downregulated), primarily involved in ribosome function and adrenergic signaling in cardiomyocytes (**Figure 3f**). For the B-vs. C group, 78 DEPs were enriched (18 upregulated and 60 downregulated) (**Figure 3i**), with dominant pathways including the citrate cycle (TCA cycle), dopaminergic synapse, insulin signaling pathway, alcoholism, insulin resistance, and plant–pathogen interactions.

**Figure 4:**
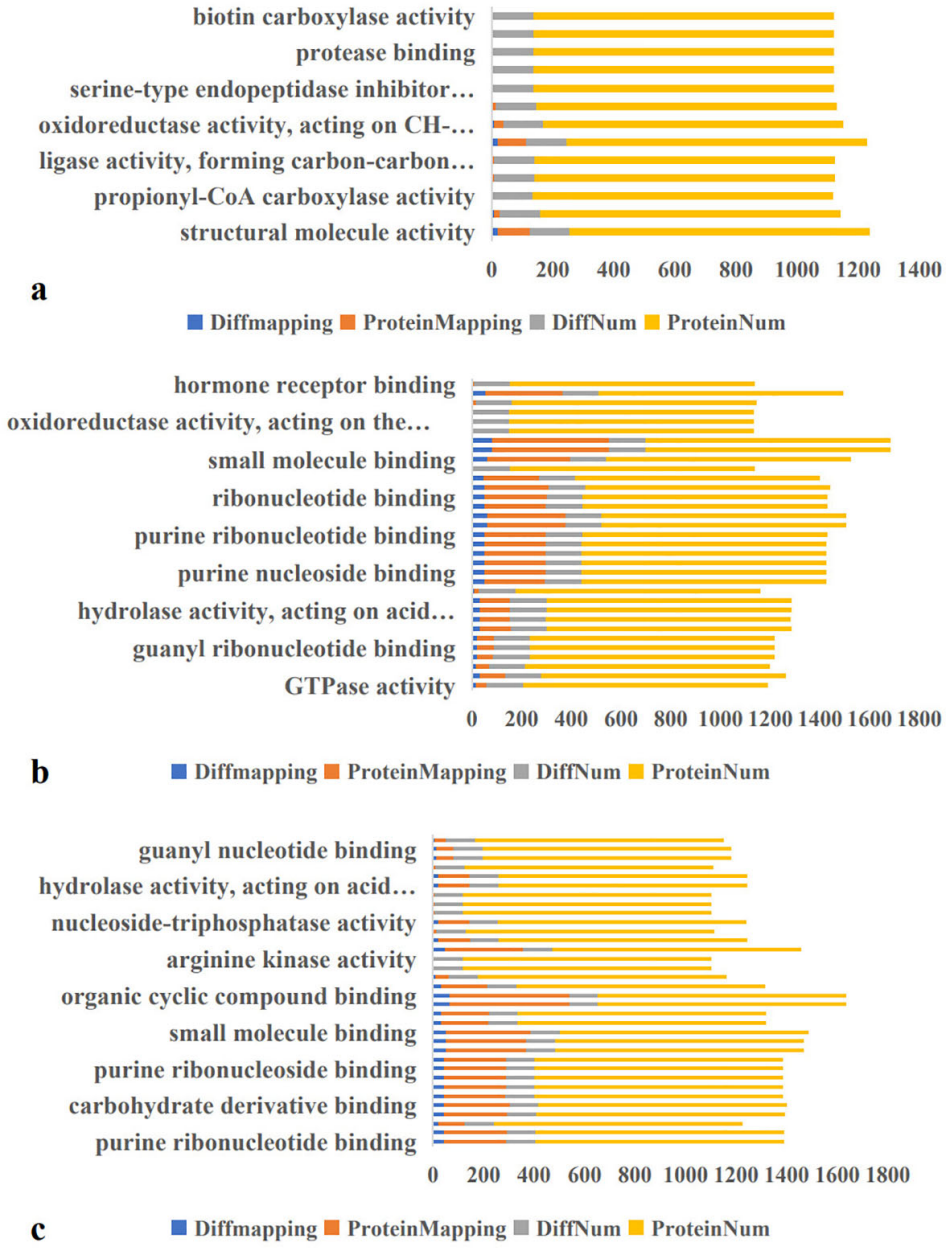
Molecular Function Enrichment in A-vs-B, A-vs-C, and B-vs-C. Diffmapping (blue), ProteinMapping (orange), DiffNum (gray), and ProteinNum (yellow). DiffMapping is the number of differentially expressed proteins in a GO term, ProteinMapping is the number of quantitative proteins in a GO term, DiffNum is the number of differentially expressed proteins in all GO terms, and ProteinNum is the number of quantitative proteins in all GO terms.

Molecular function enrichment analysis of 983 proteins identified 131 DEPs in the A-vs-B group, comprising 78 upregulated and 53 downregulated proteins (**Figure 4a**). The top two enriched molecular functions were structural molecule activity and oxidoreductase activity.

In the A-vs-C group, 146 DEPs were identified—56 upregulated and 90 downregulated (**Figure 4b**). These DEPs were primarily enriched in structural molecule activity, nucleotide binding, nucleoside phosphate binding, ribonucleoside binding and ribonucleoside binding. For the B-vs-C group, 115 DEPs were detected, including 26 upregulated and 89 downregulated proteins (**Figure 4c**). The most significantly enriched molecular functions were heterocyclic compound binding and organic cyclic compound binding.

Functional enrichment–based clustering of protein groups was performed across cellular components, molecular functions, biological processes, domains, and KEGG pathways in all three comparison groups.

**Figure 5:**
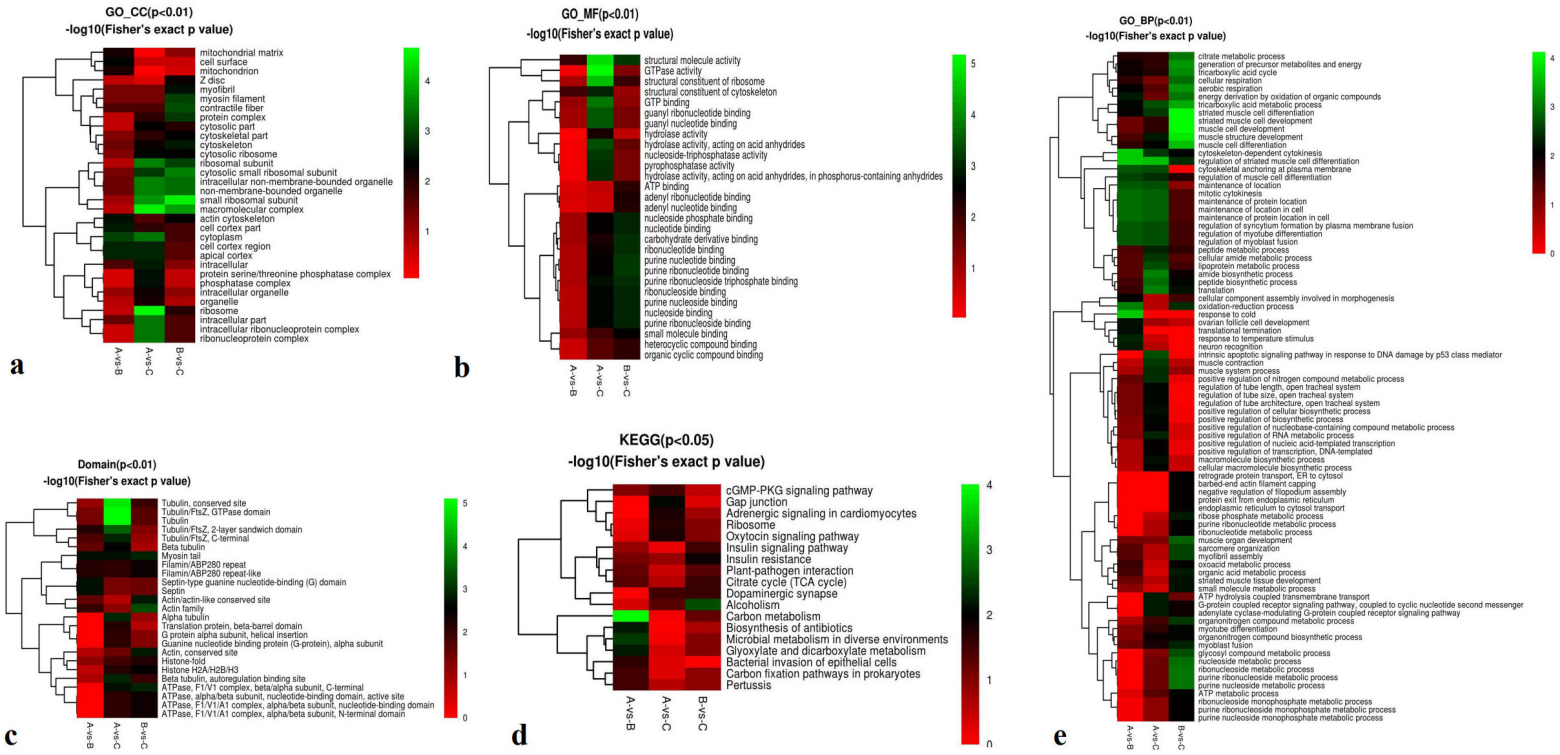
Functional enrichment-based clustering for protein groups in cellular components, molecular function, biological processes, domains, and KEGG clusters of A-vs-B, A-vs-C, and B-vs-C. From red to green (score 0-5), it means the KEGG (P<0.05 or P<0.01) -log10 (Fisher’s exact p -value).

First, cellular component analysis revealed significant differences in the ribosome and macromolecular complex in the A-vs.-C group (Fisher’s exact p -value, P < 0.01), and in the small ribosomal subunit in the Bvs. C group (P<0.01) (**Figure 5a**).

Second, molecular function analysis showed significant enrichment in structural molecule activity, GTPase activity, and structural constituents of ribosomes in the Avs. C group (P < 0.01) (**Figure 5b**). Third, domain enrichment results indicated significant differences in tubulin-related domains, including the conserved tubulin site, Tubulin/FtsZ, and GTPase domains, also in the Avs. C group (P < 0.01) (**Figure 5c**). KEGG pathway analysis revealed significant enrichment in carbon metabolism in the A vs. B group (*P* < 0.05) (**Figure 5d**). Finally, biological process analysis showed significant enrichment in striated muscle cell differentiation and development, muscle cell development, and muscle structure development in the Bvs. C group (P < 0.01) (**Figure 5e**).

### 3.4 Phosphoproteomics and phosphorylated proteins

**Figure 6:**
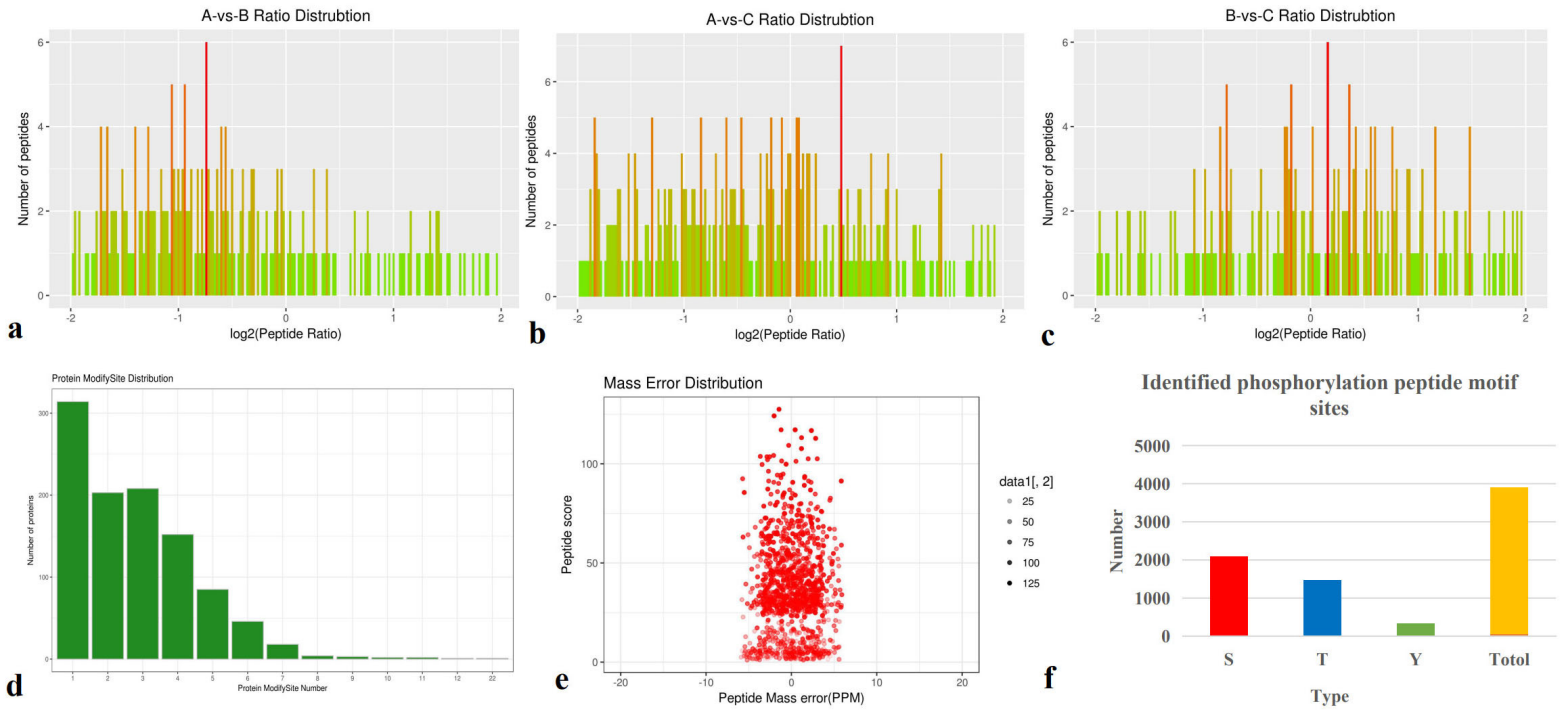
Total identified phosphorylation proteins. **(a-c)** Phosphorylation Protein Ratio Distribution of A-vs-B, A-vs-C, and B-vs-C; **(d)** Identified Phosphorylated Peptides Distribution; **(e)** Mass Error Distribution; **(f)** Identified phosphorylation motif sites: Serine (S), Threonine (T), and Tyrosine (Y).

A total of 2,914 phosphorylation modification sites and 1,039 phosphorylated proteins were identified across the three pairwise comparisons (A-vs-B, A-vs-C, and B-vs-C) (**Figures 6a–c**). The identified phosphorylation motif sites for serine (Ser), threonine (Thr), and tyrosine (Tyr) residues are shown in **Figure 6**.

Notably, these phosphorylation sites are involved in the regulation of key signaling pathways, including ubiquitin-mediated proteolysis and Ras signaling. Phosphorylation predominantly occurred on the side chains of Ser, Thr, and Tyr residues in substrate proteins, with 1,558 Ser sites (25 motif types), 1,144 Thr sites (19 motif types), and 209 Tyr sites (8 motif types) identified (**Figure 6f**).

Differentially expressed phosphorylation proteins (DEPPs) were identified based on a protein quantification threshold of fold change ≥ 1.2 and p-value ≤ 0.05. Functional classification analysis included cellular components, molecular functions, biological processes, and subcellular localization.

**Figure 7:**
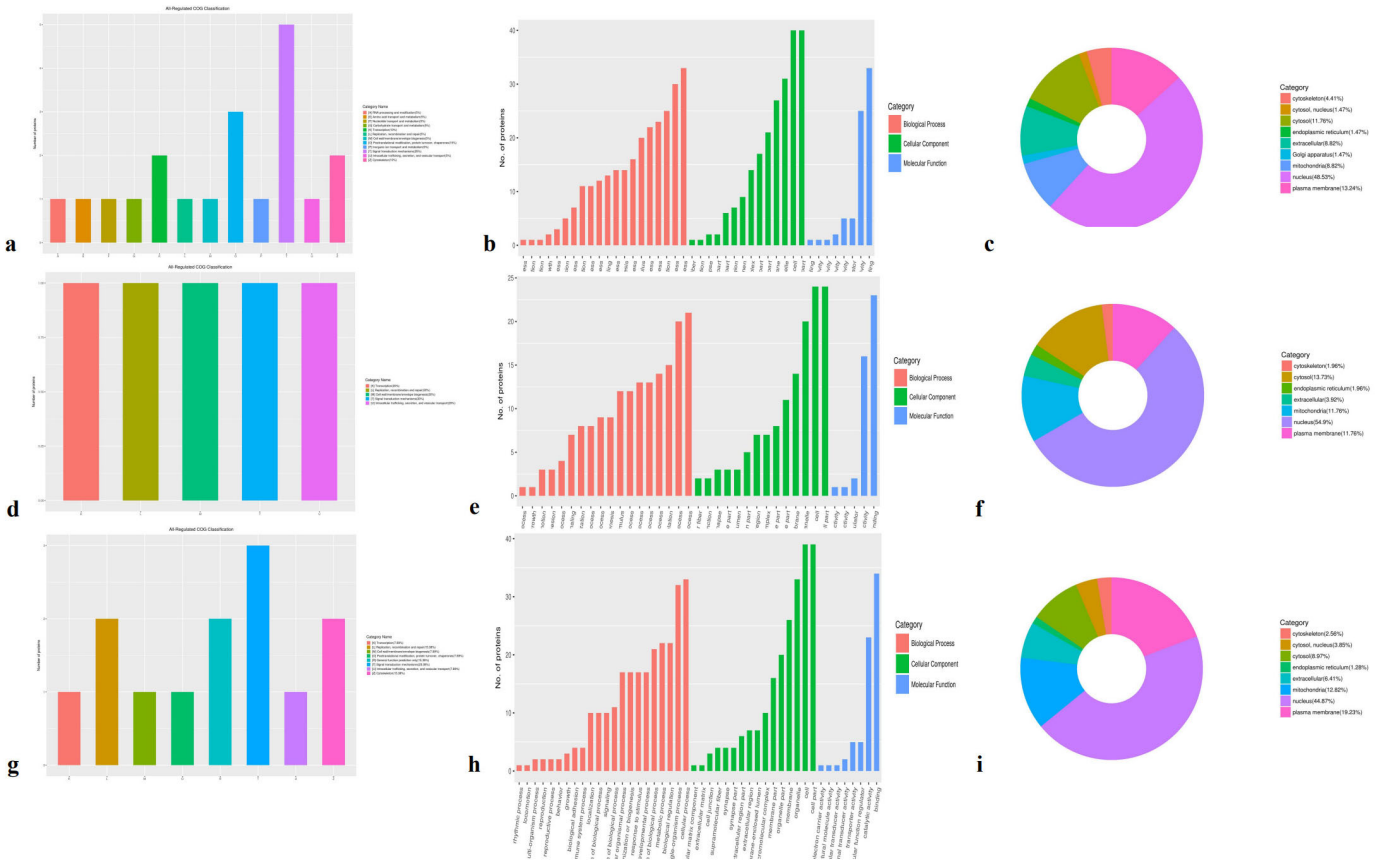
COG classification of differentially expressed phosphorylation proteins in cellular components, molecular function, biological processes, and subcellular location in three species. **(a, d, g)** All regulated COG classification in A -vs- B, A -vs- C, B -vs- C, respectively; **(b, e, h)** The category in cellular components, molecular function, and biological processes in A -vs- B, A vs. C, and B -vs- C, respectively; **(c, f, i)** The category in subcellular location in A -vs- B, A vs. C, and B -vs- C, respectively.

DEPPs were primarily involved in signal transduction mechanisms (20.0%–25.0%) and in posttranslational modification, protein turnover, and chaperones (7.69%–15.0%) across the A-vs-B, 547 A-vs-C, and B-vs-C groups (**Figures 7a, d, g**). Functional classification revealed distinct differences among the three comparison groups. These DEPPs participated in cellular components such as cells, organelles, and membranes (green columns in **Figures 7b, e, h**) and were functionally associated with binding and catalytic activity (blue columns in **Figures 7c, f, i**).

Biological process classification indicated that most DEPPs were involved in cellular processes and single-organism processes (orange columns). Subcellular localization analysis showed that DEPPs were predominantly located in the nucleus (44.87%–54.90%), mitochondria (8.82%–12.82%), and plasma membrane (11.76%–19.23%). The majority of DEPPs localized to the nucleus and were associated with phosphorylation-mediated signal transduction.

### 3.5 Phosphoproteomics of DEPs

**Figure 8:**
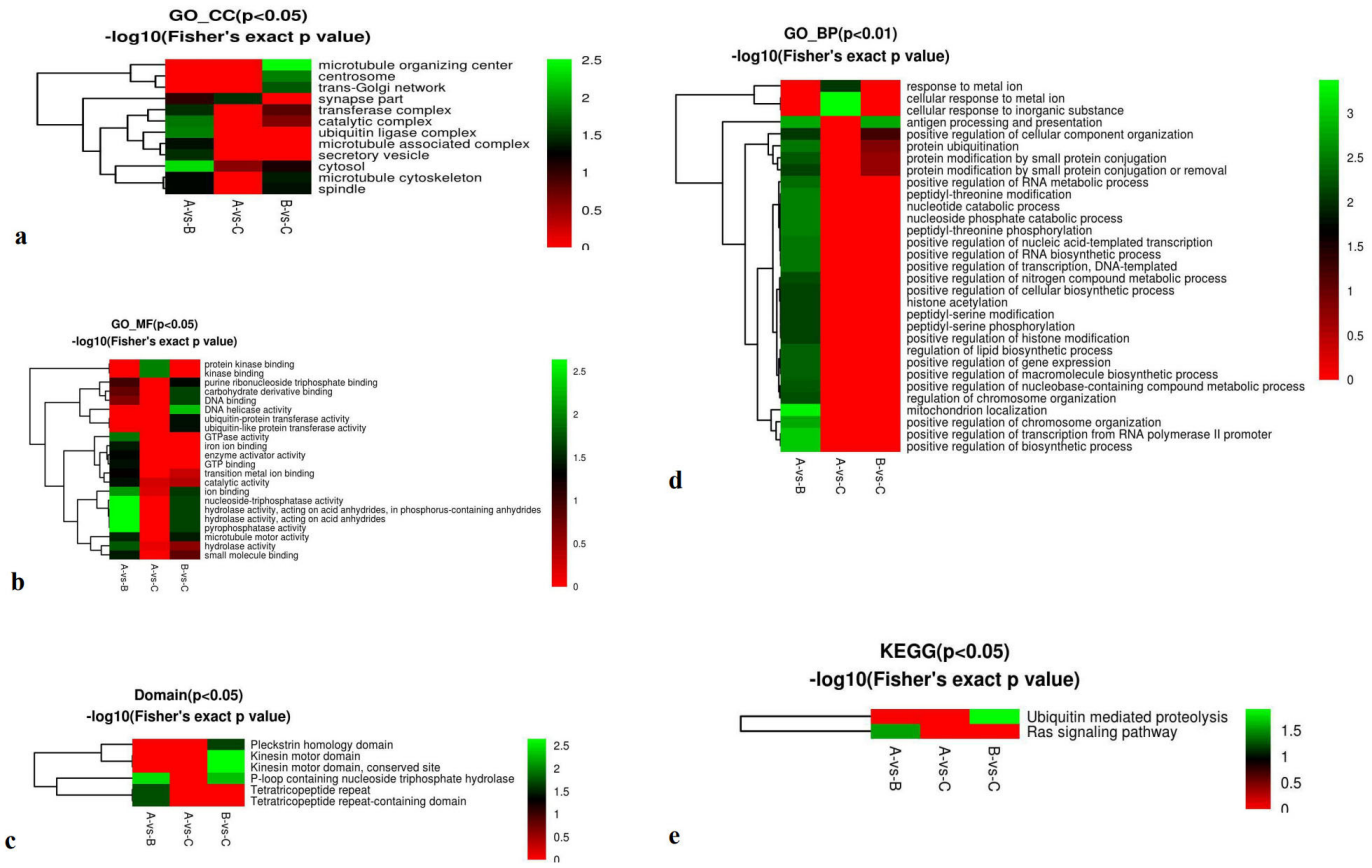
Phosphoproteomics cluster in cellular components, molecular function, biological processes, domains, and KEGG cluster of A-vs-B, A-vs-C, and B-vs-C. From red to green (score 0-5), it means the KEGG (P<0.05 or P<0.01) -log10(Fisher’s exact p value). KEGG Annotation: KEGG connects known information on molecular interaction networks, such as pathways and complexes (the “Pathway” database); information about genes and proteins generated by genome projects (including the gene database); and information about biochemical compounds and reactions (including compound and reaction databases).

Functional enrichment–based clustering of phosphorylation-associated DEPs was analyzed across cellular components, 596 molecular functions, biological processes, protein domains, and 597 KEGG pathways in the three comparison groups. Cellular 598 component analysis showed significant enrichment in the cytosol 599 for the A-vs-B group (P < 0.05) and in the microtubule organizing 600 center for the B-vs-C group (P < 0.05) (**Figure 8a**). Molecular 601 function analysis revealed significant differences in nucleoside-triphosphatase activity, hydrolase activity acting on acid anhydrides, phosphorus-containing anhydrides, and pyrophosphatase activity in the A-vs-B group (P < 0.05) 605 (**Figure 8b**). Domain enrichment analysis showed significant involvement of P-loop-containing nucleotide-binding domains in the A-vs-B group, and kinesin motor domains and their conserved sites in the B-vs-C group (P < 0.05) (**Figure 8c**). Biological process analysis indicated significant enrichment in mitochondrial localization in the A-vs-B group, as well as cellular responses to metal ions and inorganic substances (P < 0.01) (**Figure 8d**). KEGG pathway enrichment revealed significant differences in the Ras signaling pathway in the Avs. B group and in ubiquitin-mediated proteolysis in the B-vs-C group (P < 0.05) (**Figure 8e**).

## 4 Discussion

The diversity of freshwater mollusks in China has been well documented, including *Cipangopaludina chinensis* (*C. chinensis*), *Cipangopaludina cathayensis* (*C. cathayensis*), *Cipangopaludina yunnaneusis* (*C. yunnaneusis*), *Cipangopaludina menglaensis* (*C. menglaensis*), *Nodularia douglasiae* (*N. douglasiae*), *Bellamya aeruginosa* (*B.aeruginosa*), and *Radix auricularia* (*R. auricularia*) (Zhao et al., 2022; Zhou et al., 2022b). Previous studies have shown that specie differences can lead to proteomic variation under specific conditions (Russomanno et al., 2023; Paulus et al., 2022; Grabowsky et al., 2023). In the present study, we compared the proteomic and phosphoproteomic profiles of three freshwater mollusk species. Several novel proteins and phosphorylated proteins were identified and predicted to play key roles in molecular functions, biological processes, and signaling pathways. The aim of this study was to explore differential protein expression among *C. chinensis*, *R.auricularia*, and *N.douglasiae*. A total of 1,382 proteins, 2,914 modified sites, and 1,039 phosphorylated proteins were identified across the pairwise comparisons (*C. chinensis* vs. *R. auricularia*, *C. chinensis-vs-N douglasiae*, and *R. auricularia* vs. *N. douglasiae*). Our findings may provide valuable insights into the biological characteristics of these species and their potential susceptibility to microbial infections.

Whether mollusks found in nature can serve as safe and sustainable food sources remains a topic of ongoing discussion. Freshwater gastropods and bivalves have been consumed since at least the Mid–Late Neolithic period (ca. 5600–4500 BP) (Li et al., 2013). Today, species such as *C.chinensis* and *Unio douglasiae* (*U.douglasiae*) are still used as food or feed additives for both humans and animals, commonly found in rivers, reservoirs, rice fields, and experimental aquaculture systems (Li et al., 2013).

However, while freshwater mollusks can serve as a valuable protein source, they may also pose potential health risks. These include microbial co-infections with the host (Chen et al., 2021), latent parasitic infections (Chung et al., 1998; Juhász et al., 2022), and the bioaccumulation of heavy metals (Fang et al., 2001). However, there are still differing opinions on whether naturally sourced or farmed mollusks are more suitable for consumption. Some consumers prefer wild-caught mollusks for their distinctive flavor compared to cultivated varieties.

Notably, our proteomic results microbial metabolism in diverse environments revealed signatures of microbial metabolism in diverse environments across all three species, highlighting their complex interactions with environmental microbes and their potential as both a nutritional source and a model for ecological and food safety studies.

In the context of carbon neutrality, microbes and plants have received significant attention for their ability to store carbon (Pradeep Ram et al., 2016; Li et al., 2023; Song et al., 2024; Reis et al., 2022; Wang et al., 2023), whereas animals have largely been overlooked due to their relatively lower biomass and more limited distribution. However, a growing body of research indicates that the role of animals in regulating greenhouse gas emissions has been underestimated (Schmitz et al., 2018; Clare et al., 2019). According to a report by a research team at Yale University, animals can influence biogeochemical processes by 15% to 250% (Schmitz et al., 2018). In some regions, the scale of carbon uptake or release from specific animal species or groups may even rival fossil fuel emissions in the same region (Schmitz et al., 2018). Our findings revealed significant differences in carbon metabolism–related signaling pathways in the *C. chinensis*-vs.-*R.auricularia* comparison group (Gorman et al., 2023; Lind et al., 2017; Nissa et al., 2023). Notably, KEGG enrichment and functional clustering analyses showed that a substantial number of DEPs were involved in carbon metabolism, suggesting that these signaling pathways play a key role in the metabolic processes of freshwater mollusks.

Interestingly, microbial metabolism was also detected in our study, particularly in the *C. chinensis*-vs.-*R. auricularia* group. While we cannot fully exclude the possibility of microbial infection in natural hosts, it is important to consider that the growth and developmental environments of freshwater mollusks naturally contain a wide variety of microorganisms, including viruses, bacteria, and parasites (Lavelle and Sokol, 2020; Fiorucci et al., 2021; Gabriel and Ferguson, 2023; Wypych et al., 2021). Both *C. chinensis* and *R. auricularia* are univalve mollusks widely distributed in rivers and lakes. Notably, *R.auricularia* may pose a greater microbial risk in freshwater ecosystems due to its broader environmental exposure and potential to harbor pathogens (Schell et al., 2017; Juhász et al., 2022; Chung et al., 1998).

Phosphorylation plays a critical role in host cell signaling pathway transduction (Li et al., 2022; Chen et al., 2024; Bachtell et al., 2002; Heinrich et al., 1998). Our research showed that majority of DEPPs (20.0%–25.0%) were involved in signal transduction mechanisms. Moreover, these DEPPs were primarily localized to the nucleus (44.87%–54.90%), suggesting that the three freshwater species may preferentially mediate signaling pathways in the nucleus and cytoplasm. Generally, phosphorylation is a common form of post-translational modification (PTM) involving the addition of phosphate groups to proteins, usually catalyzed by kinases. Prior studies have shown that phosphorylation mainly occurs on three amino acid residues: serine (Takai et al., 2018; León-Vaz et al., 2021; Chen et al., 2013; Li et al., 2020), threonine (Hou et al., 2022), and tyrosine (Birkelund et al., 1994; Chen et al., 2017). In our study, several novel DEPPs and phosphorylation sites were identified and quantified in *C. chinensis*, *R.auricularia*, and *N.douglasiae*, which hold significance for understanding regulatory processes in freshwater mollusks.

Previous studies have reported the involvement of ubiquitin mediated proteolysis pathways in freshwater species. For example, transcriptome analysis revealed that the ubiquitin-mediated proteolysis pathway plays a key role in metabolism during testicular differentiation in *C. carpio* (Zhao et al., 2022). Skeletal muscle protein degradation, mediated through the ubiquitin proteasome system, is also crucial for energy supply, particularly under environmental stress such as crowding (Valenzuela et al., 2020). High-throughput RNA-seq analysis showed that upregulated transcripts in rainbow trout skeletal muscle were predominantly associated with ubiquitin -mediated proteolysis during *Flavobacterium psychrophilum* infection (Rivas-Aravena et al., 2019). Similarly, in *Procambarus clarkii*, oxidative stress from heavy metal exposure activated ubiquitination enzymes and proteasomes (Jiao et al., 2019). In our phosphoproteomics analysis, KEGG enrichment results revealed that the ubiquitin -mediated proteolysis pathway plays an important role in the *R.auricularia*vs. *N.douglasiae* comparison group, highlighting its relevance in stress response and protein turnover in freshwater mollusks.

In addition to ubiquitin-mediated proteolysis, Ras signaling pathways have also been implicated in freshwater species such as zebrafish, *Carassius auratus*, *Macrobrachium rosenbergii*, and *Trachemys scripta*. For example, angiotensin II within the Ras signaling pathway has been shown to regulate hemodynamic pressure overload –mediated cardiovascular pathogenesis in mammals using the zebrafish model (Joshi et al., 2021). In *Macrobrachium rosenbergii*, the Ras pathway plays a critical role in maintaining homeostasis in response to hypotonic stress at various developmental stages. Notably, in *Trachemys scripta elegans*, Ras signaling is involved in regulating spermatogenesis, inflammation, and apoptosis under salinity stress (Li et al., 2024). QTL analysis of alkaline tolerance in *C. auratus* revealed that Ras signaling may contribute to adaptation to extremely alkaline environments (Zhang et al., 2024). Our results similarly suggest that Ras signaling plays an important role in the *C. chinensis* vs. *R.auricularia* comparison group.

While our study presents some novel findings across different species, it is not without limitations. Initially, we intended to analyze the microbial communities present in freshwater mollusk carcasses. However, the volume and diversity of associated viruses, bacteria, and parasites presented a considerable workload, which could not be addressed within the current scope. We plan to explore microbial diversity in these mollusks in future studies.

Some readers may wonder why specific microbes were not tested. This is because the proteomic profiling in this study was conducted using databases relevant to mollusks (specifically snails), not databases of pathogenic microorganisms. Importantly, pathohistological observations revealed no visible tissue damage or presence of pathogenic microbes in the carcass tissues of any of the three mollusk species.

This study was primarily designed to explore the proteomic and phosphoproteomic profiles of *C. chinensis*, *R. auricularia*, and *N. douglasiae*. The results contribute new knowledge by identifying novel proteins and proteases in freshwater mollusks. Furthermore, our research highlights the importance of food safety awareness, particularly in the fishing and catering industries. Finally, bioinformatics analysis indicated that several DEPs and DEPPs may play critical roles in molecular functions, biological processes, and signaling pathways. The identification of these carcass proteins provides a new basis for understanding differential protein expression across natural environments and supports the development of novel strategies for food security in freshwater species.

## Data Availability

The datasets presented in this study can be found in online repositories. The names of the repository/repositories and accession number(s) can be found below: http://proteomecentral.proteomex-change.org, IPX0007512000; IPX0009869000; IPX0009870000.
